# The relationship between programmed cell death and vascular calcification

**DOI:** 10.3389/fcvm.2025.1549857

**Published:** 2025-07-10

**Authors:** Jie Zheng, Zhengjie Lin, Xiaolin Zhong, Jianghua Liu

**Affiliations:** The First Affiliated Hospital, Institute of Endocrinology and Metabolism, Center for Clinical Research in Diabetes, Hengyang Medical School, University of South China, Hengyang, Hunan, China

**Keywords:** vascular calcification, programmed cell death, signaling pathways, diabetic angiopathy, atherosclerosis

## Abstract

Vascular calcification (VC) is a pathological condition closely associated with a range of cardiovascular diseases, including atherosclerosis (AS), hypertension, vascular injury, and diabetic angiopathy. Programmed cell death, encompassing apoptosis, autophagy, pyroptosis and ferroptosis, plays a pivotal role in the progression of VC. These cellular processes are intricately regulated by multiple signaling pathways, such as the Wnt/β-catenin and NF-κB pathways, among others. A deeper understanding of the roles and underlying mechanisms of programmed cell death in VC could offer valuable insights for the development of innovative therapeutic strategies targeting cardiovascular diseases.

## 1 Definition, epidemiology, and treatment of VC

Vascular calcification (VC) is a pathological process characterized by the deposition and crystallization of calcium within the blood vessel walls, leading to vascular hardening and structural damage (1). This condition is frequently observed as a complication in patients with type 2 diabetes (T2D) and chronic kidney disease (CKD), and is often associated with concurrent pathological processes such as chronic inflammation and collagen proliferation (2, 3). Pathological investigations have established that VC development is closely linked to inflammatory responses, tissue repair mechanisms, and metabolic dysregulation (4). In the context of atherosclerosis (AS), VC significantly influences plaque stability. While minimal calcification may predispose plaques to rupture, extensive calcification can enhance plaque rigidity and stability (5). Epidemiological evidence indicates that VC serves as an independent risk factor for cardiovascular events and mortality in adults. The prevalence of VC demonstrates an age-dependent increase, with additional risk factors including hypercholesterolemia, hypertension, obesity, smoking, physical inactivity, and genetic predisposition (6, 7). Current clinical management of VC encompasses pharmacological interventions, interventional procedures, and surgical approaches (8). Pharmacological treatments typically involve cholesterol-lowering agents, antihypertensive medications, diuretics, and calcium channel blockers. Interventional strategies primarily include balloon angioplasty and arterial bypass grafting (9). Surgical intervention is generally reserved for cases of severe VC resulting in significant vascular stenosis and ischemia (10). Complementary non-pharmacological interventions, including dietary modification, regular exercise, and smoking cessation, have been shown to alleviate symptoms and decelerate disease progression. Given the clinical significance of VC, elucidation of its underlying mechanisms is crucial for the development of novel therapeutic strategies to mitigate its progression and associated complications.

## 2 Cell types that participate in VC

**Figure 1:**
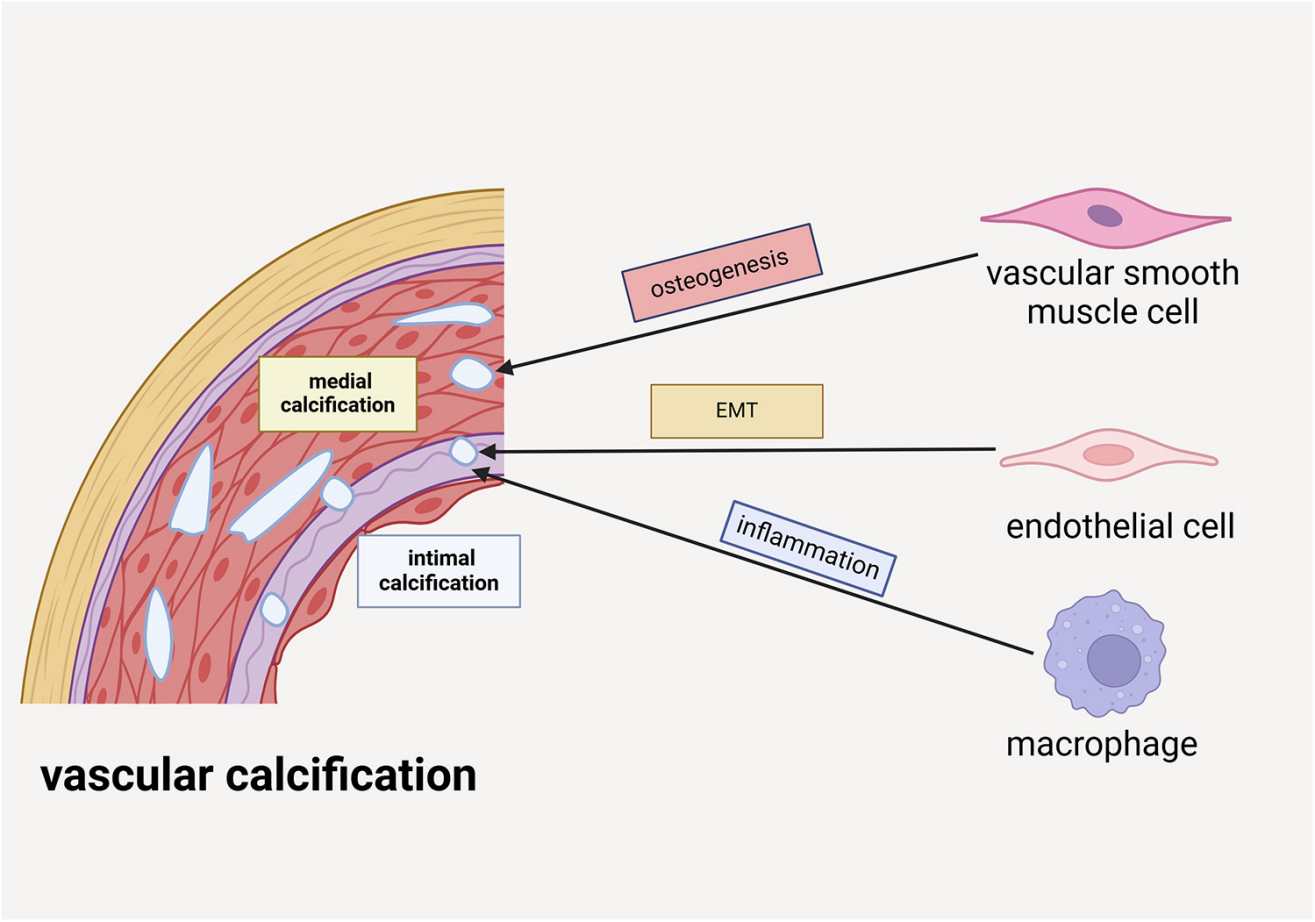
VSMCs, ECs and macrophages are involved in the formation of vascular calcification. VSMCs participate in medial calcification by undergoing osteogenic differentiation. ECs are implicated in intimal calcification through the process of EMT. Additionally, macrophages contribute to intimal calcification by driving inflammatory mechanisms.

The pathogenesis of VC involves multiple cellular components within the vascular wall, including vascular smooth muscle cells (VSMCs), endothelial cells (ECs), and macrophages, each playing distinct roles in this pathological process. Under physiological conditions, VSMCs maintain a contractile phenotype characterized by significant plasticity. However, under pathological stimuli, these cells can undergo phenotypic transition into osteoblast-like cells, initiating calcium deposition within the cellular matrix and contributing to VC progression. Notably, VSMC apoptosis precedes calcification formation and intensifies with disease progression. The apoptotic bodies derived from VSMCs possess calcium-concentrating capabilities, further facilitating calcification (11). ECs contribute to VC through endothelial-mesenchymal transition (EMT), acquiring multipotent differentiation capacity. Through activation of TGF-β and Wnt signaling pathways, these transformed ECs can differentiate into osteoblast-like cells. Under pathological conditions such as chronic inflammation and hyperglycemia, ECs release extracellular vesicle (EV) containing calcium ions and bone morphogenetic protein 2 (BMP2), which serve as nucleation sites for calcification initiation and progression (12).CKD elevated phosphate levels not only directly promote VC but also stimulate ECs to secrete tissue-nonspecific alkaline phosphatase. This enzyme catalyzes the degradation of extracellular pyrophosphate into phosphate ions, thereby eliminating its natural vascular protective effect (1). Macrophages exhibit dual roles in VC pathogenesis, with their phenotypic polarization determining their functional impact. M1 macrophages significantly promote VC through the release of pro-inflammatory cytokines that enhance VSMCs osteogenic differentiation. Conversely, M2 macrophages exert inhibitory effects via anti-inflammatory factor secretion and phagocytic clearance of apoptotic cells. Furthermore, macrophages contribute to VC through multiple mechanisms: (1) secretion of various cytokines and osteogenic factors that promote vascular cell differentiation; (2) release of microvesicles (MVs) that serve as calcium phosphate nucleation sites; and (3) potential differentiation into osteoclast-like cells that may counteract VC development (13). The complex interplay between these cellular components and their phenotypic states underscores the multifaceted nature of VC pathogenesis. Elucidating the precise roles and regulatory mechanisms of these cellular participants is crucial for developing targeted therapeutic strategies against VC (Figure 1).

## 3 Programmed cell death plays a critical role in VC

Emerging evidence has identified apoptosis, autophagy, and pyroptosis as the principal forms of programmed cell death implicated in the pathogenesis of VC, with VSMCs and ECs serving as the primary cellular mediators (14, 15). Among these, apoptosis represents the most extensively studied form of programmed cell death in VSMCs during VC development. Apoptotic cells release matrix vesicles enriched with calcium and phosphate, which serve as direct mediators of extracellular matrix mineralization (16). In contrast to the pro-calcific effects of apoptosis, autophagy acts as a protective mechanism against VC through its catabolic function. This process prevents the accumulation of senescent cells and calcifying vesicles, thereby exerting an inhibitory effect on vascular mineralization (17). Pyroptosis, characterized by its inflammatory nature, is triggered through death receptor activation and contributes to VC progression through the release of calcifying extracellular vesicles containing calcium and phosphate ions (18, 19). Recent investigations have revealed that ferroptosis, an iron-dependent form of cell death, promotes VC through mechanisms involving oxidative stress and lipid peroxidation. These processes induce cellular damage, leading to abnormal calcium and phosphate ion deposition within the vascular wall (20). Despite these advances, the precise molecular mechanisms underlying these distinct cell death pathways and their intricate interplay in VC pathogenesis remain to be fully elucidated, warranting further comprehensive investigation.

### 3.1 Apoptosis and vascular calcification

Apoptosis represents a fundamental physiological process of programmed cell death that plays a crucial role in maintaining cellular homeostasis and tissue integrity. This process is mediated through two distinct pathways: the intrinsic and extrinsic pathways. The intrinsic pathway is initiated by intracellular apoptotic signals that induce mitochondrial outer membrane permeabilization, leading to the release of cytochrome c into the cytosol. This event subsequently triggers a cascade of enzymatic reactions involving various cytokines and caspases, ultimately resulting in apoptotic cell death. In contrast, the extrinsic pathway is activated through the recognition of extracellular death signals by specific cell surface receptors, initiating a downstream signaling cascade. Both pathways converge on the activation of executioner caspases, particularly caspases-3 and -7, which mediate the characteristic morphological changes of apoptosis. These changes include cellular fragmentation into membrane-bound apoptotic bodies, which are subsequently recognized and phagocytosed by macrophages. Under pathological conditions, particularly in the context of vascular calcification, apoptotic bodies released by VSMCs have been shown to significantly promote the calcification process (19).

#### 3.1.1 Inhibition of VSMCs apoptosis can effectively improve VC

Accumulating evidence demonstrates that pharmacological interventions targeting apoptosis can effectively attenuate VC. Traditional Chinese medicine compounds have shown promising anti-calcification effects through apoptosis modulation. Shenqi has been found to inhibit diabetic VC by suppressing the Hippo-YAP signaling pathway, thereby reducing VSMCs apoptosis and inflammation while providing vascular protection (21). Baicalin, a bioactive flavonoid extracted from Scutellaria baicalensis, significantly inhibits β-glycerophosphate-induced VSMCs apoptosis and osteogenic differentiation, consequently suppressing VC progression (22). Shuxuetong exhibits dual protective effects by inhibiting dexamethasone-induced oxidative stress-mediated apoptosis, consequently preventing both vascular calcification and osteoporosis (23). Dendrobium officinale polysaccharides demonstrate therapeutic potential through Heme Oxygenase-1 (HMOX-1) activation, effectively inhibiting high-phosphate-induced VSMCs apoptosis and inflammation, while significantly ameliorating VC in CKD mouse models (24). Ginsenoside Rb1, a major active component of Panax ginseng, exerts cardiovascular protective effects by inhibiting VSMCs apoptosis and VC under high-phosphate conditions through upregulation of the Gas6/pAkt pathway (25). Experimental studies have identified several cytokines with anti-apoptotic and anti-calcification properties. Growth Differentiation Factor 11 emerges as a potential therapeutic cytokine, effectively inhibiting both VSMCs apoptosis and osteogenic differentiation (26). Fibroblast Growth Factor 21 demonstrates protective effects by suppressing oxidative stress-induced apoptosis and VC through inhibition of the C/EBP Homologous Protein (CHOP) and caspase-12 signaling pathways (27). Protein-based interventions have also shown significant anti-calcification effects. Activation of nuclear factor erythroid 2-related factor 2 (NRF2) effectively reduces apoptosis and osteogenic gene expression in VSMCs under high-phosphate conditions, thereby inhibiting VSMCs osteogenesis and VC progression (28). Overexpression of sclerostin protein significantly reduces calcium deposition in VSMCs under calcifying conditions through apoptosis inhibition, representing a novel therapeutic direction for VC treatment (29). Polypeptide N-acetylgalactosamine transferase 3 exerts its anti-calcification effects by reducing apoptosis through inhibition of oxidative stress and the TNFR1/NF-*κ*B signaling pathway in high-phosphate conditions (30).

#### 3.1.2 Promoting apoptosis exacerbates VC

Emerging research has elucidated complex molecular mechanisms through which various biochemical factors promote VC via apoptosis induction in VSMCs. Calcium ions and aldosterone synergistically interact through the AIF-1/NF-κB signaling pathway, exacerbating both apoptosis and inflammation in VSMCs under uremic conditions, thereby accelerating VC progression (31). Parathyroid hormone contributes to VC pathogenesis by inducing oxidative stress through dual signaling pathways: the PERK-CHOP and IRE1-JNK cascades, which collectively promote VSMCs apoptosis and calcification (32). Beyond hormonal influences, various exogenous and endogenous substances have been implicated in VC progression through apoptosis modulation.Nano-Sized Hydroxyapatite has been shown to promote VSMCs apoptosis and osteogenic differentiation via activation of the JNK/c-JUN signaling pathway, thereby aggravating VC (33). 25-Hydroxycholesterol, an oxysterol derived from enzymatic cholesterol oxidation, plays a significant role in cellular signaling by promoting apoptosis and exacerbating VC through activation of the ATF4/CHOP signaling pathway (34). Metabolic factors also contribute significantly to VC pathogenesis. Glucose variability participates in VC development through oxidative stress-mediated apoptosis, potentially involving multiple molecular mediators including Wnt1, galectin-3, and BMP-2 in this process (35). Furthermore, saturated fatty acids have been demonstrated to promote VSMCs apoptosis in VC by suppressing SIRT6 expression, revealing a novel mechanism linking lipid metabolism to VC (36).

#### 3.1.3 Endothelial cell apoptosis is involved in the process of VC

ECs serve as critical regulators of vascular homeostasis, maintaining essential functions including vasodilation regulation, thrombosis inhibition, and vascular wall integrity preservation. However, ECs apoptosis can compromise vascular barrier function, thereby exacerbating VC. Homocysteine, a sulfur-containing amino acid derived from methionine and cysteine metabolism, has been implicated in vascular pathology. Professor Liu's research team demonstrated that elevated homocysteine concentrations significantly enhance ECs apoptosis rates and promote VC progression (37). ECs-derived microvesicles, particularly exosomes, play a significant role in VC through intercellular communication. Research has revealed that exosomes released by ECs under hyperglycemic conditions can induce VSMCs calcification and senescence (38). Comparative studies have shown that CKD patients exhibit significantly higher levels of endothelial-derived EVs in circulation compared to healthy individuals, suggesting that ECs are the primary source of circulating EVs in CKD. These EVs exhibit dual pathological effects: promoting VSMCs calcification *in vitro* and inducing endothelial dysfunction, characterized by increased apoptosis in human umbilical vein endothelial cells and impaired angiogenesis. These effects are mediated through the upregulation of miR-223 levels within EVs (39). Furthermore, ECs exposed to the uremic toxin indoxyl sulfate release MVs with elevated calcium content. These calcium-rich MVs promote VC by modulating the expression of inflammatory genes in VSMCs, revealing another mechanism linking uremic toxins to vascular pathology (40).

#### 3.1.4 MicroRNAs play important roles in VSMCs apoptosis and then participate in VC

Emerging evidence highlights the dual regulatory roles of microRNAs in VC through modulation of VSMC apoptosis. Certain microRNAs have been identified as promoters of VC by enhancing VSMCs apoptosis. Specifically, miR-155 overexpression exacerbates VC by inducing VSMCs apoptosis through the Akt-FOXO3a signaling pathway (41). Conversely, several microRNAs demonstrate protective effects against VC by suppressing apoptosis.MiR-146-5p exerts anti-apoptotic effects by targeting tumor necrosis factor receptor-associated factor 6 and simultaneously inhibiting VSMCs osteogenic differentiation (42). Further research has identified additional microRNAs with protective roles in VC pathogenesis. MiR-140-5p overexpression significantly inhibits β-glycerophosphate-induced VSMC apoptosis by targeting Toll-like receptor 4 (43). In diabetic models, miR-128-3p activates the Wnt pathway while suppressing ISL1 expression, thereby inhibiting both VSMCs apoptosis and osteogenic differentiation (44). Notably, the therapeutic potential of exosomal microRNAs has also been demonstrated. Mesenchymal stem cell-derived exosomes containing miR-381-3p protect against VC by inhibiting VSMC apoptosis and osteogenesis through suppression of nuclear factor of activated T cells 5 (NFAT5) expression (45). These findings collectively underscore the pivotal role of cellular apoptosis in VC pathogenesis, with microRNAs serving as critical regulators that influence disease progression through modulation of apoptotic pathways in VSMCs.

### 3.2 Autophagy and vascular calcification

Autophagy is a fundamental cellular process that plays a vital role in maintaining normal cellular functions. It involves the encapsulation of damaged organelles and proteins by autophagosomes, which are subsequently degraded and recycled through lysosomal activity (46). Recent studies have demonstrated that autophagy is essential for preserving the normal contractile phenotype of VSMCs, thereby preventing their transition to a synthetic phenotype and reducing the risk of cardiovascular diseases (47). In healthy cells, autophagy selectively removes damaged or redundant mitochondria, underscoring its critical role in maintaining mitochondrial homeostasis (48). Interestingly, during the calcification process of VSMCs, key autophagy-related genes such as Microtubule-associated protein 1 light chain 3-I (LC3-I), p62, and Insulin-like Growth Factor Binding Protein 3 (IGFBP3) are significantly upregulated, suggesting a direct involvement of autophagy in VC (49). However, when lysosomal function in VSMCs is impaired and autophagy is inhibited, the progression of VC is markedly exacerbated (50).

#### 3.2.1 AMPK-mediated autophagy regulates VC

It is widely acknowledged that the AMP-activated protein kinase (AMPK)-related signaling pathway plays a pivotal role in regulating the initiation and progression of autophagy. Extensive research has demonstrated that AMPK-mediated activation of autophagy significantly contributes to the inhibition of VC (51). Metformin, a first-line medication for diabetes management, has been shown by several studies to reverse VSMC calcification through the AMPK-eNOS-NO and AMPK-RANKL signaling pathways, underscoring its potential in vascular protection (52, 53). Additionally, other therapeutic agents, such as policosanol, have been reported to suppress VC via the AMPK signaling pathway (54). Furthermore, melatonin has been found to enhance autophagy in VSMCs by upregulating the AMPK/mTOR/ULK1 signaling pathway, thereby effectively ameliorating β-glycerophosphate (β-GP)-induced calcification (55). Investigating the regulatory role of AMPK in autophagy may provide novel strategies for the prevention and treatment of VC in the future.

#### 3.2.2 RNA participates in vascular calcification by regulating autophagy

RNA not only plays a regulatory role in apoptosis but is also actively involved in the modulation of autophagy. Professor Liu's discovered that the expression of long non-coding RNA (LncRNA) SNHG1 is significantly downregulated in VSMCs under high glucose conditions. Further investigations revealed that SNHG1 regulates autophagy by promoting the expression of Blhe40 mRNA, which in turn modulates atg10, thereby exerting a protective effect on VSMCs (56). In addition to LncRNAs, microRNAs also play a crucial role in the regulation of autophagy. For example, under high phosphorus conditions, miR-30b has been shown to regulate autophagy by targeting BECN1 (57). Overexpression of miR-30b enhances autophagy in VSMCs and inhibits VC through modulation of the mTOR signaling pathway (58). Similarly, under high glucose conditions, the expression of miR-32 in exosomes secreted by macrophages is significantly upregulated. miR-32 suppresses autophagy in VSMCs and promotes VC by targeting Mef2d (59).

#### 3.2.3 Autophagy can improve VC by inhibiting apoptosis

Autophagy is essential for maintaining the normal function and phenotype of VSMCs by clearing damaged organelles and misfolded proteins (14). Currently, a growing number of studies indicate that certain drugs protect VSMCs by enhancing autophagy and reducing cell apoptosis. For instance, Iron citrate can inhibit calcium deposition in VSMCs under high phosphorus conditions through the GAS 6/AXL signaling pathway, promoting autophagy and suppressing cell apoptosis (60). Rosuvastatin effectively inhibits Platelet-Derived Growth Factor(PDGF)-induced VSMCs apoptosis by activating autophagy and suppressing P38 expression, thus providing effective protection for VSMCs (61). However, knocking down Heat Shock Protein Family B Member 1(HSPB1) in VSMCs reduces the expression of autophagy-related proteins, significantly enhancing cell apoptosis (62).

### 3.3 Pyroptosis and vascular calcification

Upon recognition of extracellular signals, cells assemble and activate inflammasomes, a process that serves as the initiating event of pyroptosis. Following activation, inflammasomes modify Cysteine Aspartate-Specific Protease-1 (CASPASE-1), which in turn cleaves Gasdermin D (GSDMD), a key pyroptosis effector protein. The cleaved GSDMD is then inserted into the cell membrane, forming pores that disrupt membrane integrity, ultimately resulting in cell rupture and death (19).

#### 3.3.1 Inhibiting pyroptosis can effectively suppress VC

Pang et al. demonstrated that pyroptosis plays a significant role in VC, and its inhibition effectively attenuates calcification. Irisin, an exercise-induced hormone-like protein, is known to contribute to cardiovascular health protection. Their study revealed that irisin activates autophagy to suppress NOD-like receptor thermal protein domain-associated protein 3 (NLRP3)-mediated pyroptosis, thereby preventing VC (63). Additionally, VX-765, a selective caspase-1 inhibitor, has been shown to effectively suppress pyroptosis in VSMCs under high-phosphate conditions, significantly reducing aortic calcification (64). Calcium deposition in atherosclerotic plaques is also a component of VC, and pyroptosis exacerbates plaque destabilization and increases plaque area. Growing evidence suggests that inhibiting pyroptosis can effectively mitigate plaque formation. Zhang et al. demonstrated through animal experiments that polydatin reduces plaque burden and stabilizes plaques by activating autophagy and inhibiting NLRP3-mediated pyroptosis (65). Similarly, apigenin has been found to inhibit macrophage pyroptosis by suppressing NLRP3 inflammasome activation and oxidative stress, thereby reducing atherosclerotic plaque area (66). Cui et al. reported that fucoxanthin suppresses pyroptosis by modulating the PI3K/AKT and TLR4/NF-κB signaling pathways, effectively attenuating atherosclerotic plaque formation (67). Salvianolic acid A has been shown to inhibit high-glucose-induced endothelial cell pyroptosis by downregulating the PKM2/PKR signaling pathway, thereby improving plaque stability in diabetic mice and providing vascular protection (68). Furthermore, exosomes secreted by cells can also effectively inhibit pyroptosis. For example, miR-199a-5p in macrophage-derived exosomes inhibits endothelial cell pyroptosis by downregulating SMARCA4 expression, leading to a significant reduction in atherosclerotic plaque area (69).

#### 3.3.2 Promoting pyroptosis can exacerbate VC

Studies have demonstrated that the promotion of pyroptosis can significantly exacerbate calcium deposition in atherosclerotic plaques. Genetic material, particularly RNA, plays a pivotal role in driving pyroptosis and facilitating plaque formation. lncRNA Gaplinc has been shown to promote EC pyroptosis by binding to the transcription factor Sp1. Knockout of Gaplinc significantly inhibits EC pyroptosis induced by oxidized low-density lipoprotein (ox-LDL) (70). Similarly, Linc00657 enhances EC pyroptosis under ox-LDL conditions by sequestering miR-106b-5p, leading to upregulated expression of thioredoxin-interacting protein (TXNIP) and subsequent enlargement of plaque area (71). Emerging evidence indicates that proteins and enzymes also contribute to atherosclerotic plaque formation by promoting pyroptosis. For example, Cathepsin B facilitates the nuclear translocation of NF-*κ*B, thereby inducing cell pyroptosis. Furthermore, tissue protease B has been shown to significantly accelerate plaque formation, while its inhibition results in a marked reduction in plaque burden (72). Additionally, the expression of IQ motif-containing GTPase-activating protein 1 (IQGAP1) is notably elevated in atherosclerotic plaques. IQGAP1 protein activates NLRP3-mediated EC pyroptosis by upregulating the cGAS-STING pathway, thereby exacerbating plaque formation (73).

### 3.4 Ferroptosis and vascular calcification

Ferroptosis, a distinct form of regulated cell death, is characterized by the accumulation of iron, Reactive Oxygen Species(ROS), and phospholipids containing unsaturated fatty acids. This process is driven by the peroxidation of intracellular phospholipids, which ultimately leads to cell death. Emerging evidence in recent years has increasingly demonstrated the significant involvement of ferroptosis in the pathogenesis of cardiovascular diseases and its crucial role in immune surveillance mechanisms (5).

#### 3.4.1 Inhibiting VSMCs ferroptosis effectively suppresses VC

Emerging studies have established a significant association between ferroptosis and VC. Notably, Ye et al. demonstrated that ferroptosis plays a crucial role in high phosphate- and calcium-induced VC. Their findings revealed that inhibition of key ferroptosis regulators, including solute carrier family 7 member 11 (SLC7A11) and glutathione peroxidase 4 (GPX4), markedly exacerbates VC progression (74). Palmitic acid, a saturated fatty acid ubiquitously present in animal and plant fats, has been implicated in cardiovascular risk when consumed in excess. Mechanistically, palmitic acid promotes VSMCs osteogenic differentiation and enhances periostin expression in the extracellular matrix. These changes are associated with downregulation of SLC7A11 and GPX4, thereby increasing VSMCs susceptibility to ferroptosis. Importantly, metformin has been shown to effectively counteract PA-induced VC and VSMCs ferroptosis (75). Furthermore, the cytokine fra-1 (FOSL1) exhibits upregulated expression in VSMCs under high phosphate and calcium conditions. Genetic knockout studies have demonstrated that FOSL1 deletion increases SLC7A11 expression, subsequently inhibiting reactive oxygen species generation and calcification processes, suggesting FOSL1's involvement in VC through ferroptosis regulation (20). The role of ferroptosis extends to AS development, where excessive iron promotes macrophage lipid uptake and foam cell formation. Experimental evidence indicates that ferroptosis contributes to atherosclerotic plaque formation through multiple mechanisms. For instance, cigarette tar has been shown to increase plaque area by activating NF-κB, which upregulates hepcidin while downregulating the ferroptosis regulators SLC7A11 and GPX4, ultimately promoting macrophage ferroptosis and plaque expansion (76) Similarly, hyperuricemia significantly downregulates SLC7A11 and GPX4 expression in plaques, promoting macrophage ferroptosis and aggregation, which contributes to increased plaque area (77). Additionally, Jak2VF-induced red blood cells have been found to promote ROS and lipid peroxidation accumulation, driving macrophage ferroptosis and expanding the necrotic core area of atherosclerotic plaques (78).

#### 3.4.2 Inhibiting ECs ferroptosis can effectively suppresses VC

**Figure 2:**
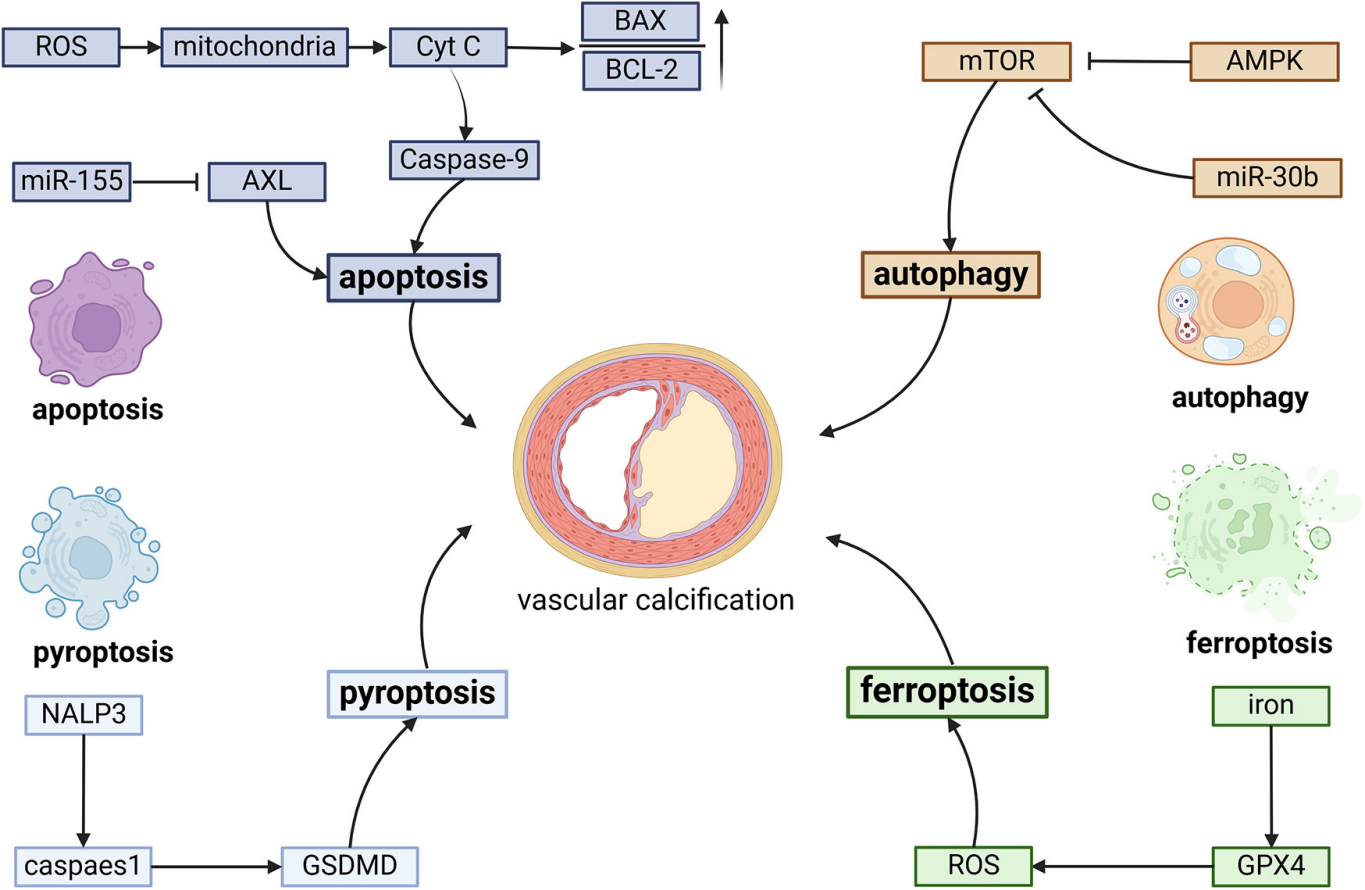
The fundamental molecular pathways involved in programmed cell death during vascular calcification include several key processes. Apoptosis is triggered by caspase-9 activation, an elevated BAX/Bcl-2 ratio, and suppression of AKT signaling. Autophagy is promoted through the inhibition of mTOR, regulated by AMPK and miR-30b. Pyroptosis is initiated by the NLRP3 inflammasome, which activates GSDMD. Excessive iron accumulation can trigger ferroptosis through the excessive generation of ROS.

Iron overload plays a significant role in cardiovascular pathology by promoting ROS generation, which damages ECs and contributes to atherosclerotic plaque formation. N-acetylneuraminic acid (Neu5Ac), a nine-carbon monosaccharide, has recently emerged as a critical factor in cardiovascular diseases. Mechanistically, Neu5Ac promotes ferroptosis and induces inflammatory damage in ECs through downregulation of the ferroptosis regulator solute carrier family 3 member 2 (SLC3A2), ultimately leading to increased plaque area and exacerbation of necrotic cores (79). Therapeutic interventions targeting EC ferroptosis have shown promising results in preventing plaque progression. Hydroxysafflor yellow A (HSYA), a valuable pharmaceutical compound extracted from Carthamus tinctorius L. (safflower) of the Asteraceae family, demonstrates significant anti-atherosclerotic effects. HSYA inhibits miR-429 expression, resulting in upregulation of SLC7A11 and subsequent suppression of ECs ferroptosis, effectively reducing atherosclerotic plaque formation (80). Another promising therapeutic agent is icariin, a bioactive flavonoid glycoside derived from Epimedium species. Icariin exerts cardiovascular protective effects by activating autophagy pathways, thereby inhibiting EC ferroptosis and reducing plaque area (81). Endocrine regulation also plays a crucial role in modulating EC ferroptosis. Estradiol has been shown to upregulate nuclear factor erythroid 2-related factor 2 (NRF2) and GPX4 expression, effectively inhibiting EC ferroptosis and improving lipid accumulation in atherosclerotic plaques in menopausal mouse models (82). Furthermore, Hu et al. demonstrated that vitamin D receptor overexpression protects against atherosclerosis by inhibiting EC ferroptosis through AMPK signaling pathway, resulting in reduced plaque area and lipid deposition (83) (Figure 2).

## 4 Conclusion

Cardiovascular diseases continue to represent the predominant cause of global mortality, with VC significantly contributing to the increased incidence of cardiovascular complications. Consequently, investigating the underlying mechanisms of VC holds substantial importance for public health. Our comprehensive review demonstrates that programmed cell death serves as a fundamental pathophysiological mechanism in VC progression. Specifically, apoptosis, autophagy, pyroptosis, and ferroptosis exert distinct regulatory effects on VC pathogenesis. These findings underscore the critical importance of elucidating the complex interplay between various forms of programmed cell death and VC, which may provide valuable insights for developing therapeutic strategies against VC.

## Glossary

**Table:**

Abbreviation	Full name	Notes
VC	vascular calcification	The deposition of calcium in vascular tissues
AS	atherosclerosis	A disease characterized by plaque buildup in arteries
T2D	type 2 diabetes	A metabolic disorder characterized by insulin resistance
CKD	chronic kidney disease	Progressive loss of kidney function over time.
VSMCs	vascular smooth muscle cells	Cells responsible for vascular contraction and matrix secretion
ECs	endothelial cells	Cells lining the interior surface of blood vessels.
EMT	endothelial-mesenchymal transition	Process where endothelial cells transform into mesenchymal cells.
EV	extracellular vesicle	Membrane-bound vesicles released by cells for intercellular communication.
BMP2	bone morphogenetic protein 2	A protein involved in bone formation and vascular calcification
MVs	microvesicles	Small extracellular vesicles involved in cell signaling
CHOP	C/EBP Homologous Protein	A transcription factor involved in endoplasmic reticulum stress.
NRF2	nuclear factor erythroid 2-related factor 2	A transcription factor regulating antioxidant response
HMOX-1	Heme Oxygenase-1	An enzyme that degrades heme and has anti-inflammatory effects
NFAT5	nuclear factor of activated T cells 5	A transcription factor involved in osmotic stress response.
IGFBP3	Insulin-like Growth Factor Binding Protein 3	A protein that binds to insulin-like growth factors.
LC3-I	Microtubule-associated protein 1 light chain 3-I	A marker of autophagosome formation.
*β*-GP	*β*-glycerophosphate	A compound used to induce vascular calcification *in vitro*
LncRNA	long non-coding RNA	Non-coding RNA molecules involved in gene regulation
HSPB1	Heat Shock Protein Family B Member 1	A small heat shock protein with anti-apoptotic function
CASPASE-1	Cysteine Aspartate-Specific Protease-1	An enzyme involved in pyroptosis and inflammation
GSDMD	Gasdermin D	A protein that forms pores in the cell membrane during pyroptosis
NLRP3	NOD-like receptor thermal protein domain-associated protein 3	A component of the inflammasome complex
ox-LDL	oxidized low-density lipoprotein	A modified form of LDL involved in atherosclerosis
IQGAP1	IQ motif-containing GTPase-activating protein 1	A scaffold protein involved in cell signaling
SLC7A11	solute carrier family 7 member 11	A cystine/glutamate transporter involved in ferroptosis
GPX4	glutathione peroxidase 4	An enzyme that protects cells from lipid peroxidation
Neu5Ac	N-acetylneuraminic acid	A sialic acid involved in cell signaling and recognition
SLC3A2	solute carrier family 3 member 2	A component of the cystine/glutamate transporter system
HSYA	Hydroxysafflor yellow A	A natural compound with anti-inflammatory and antioxidant properties
AMPK	the AMP-activated protein kinase	A cellular energy sensor regulating metabolism
ROS	Reactive Oxygen Species	Molecules involved in oxidative stress and signaling.
YAP	Yes-Associated Protein	A transcriptional regulator in the Hippo signaling pathway.
NAF2	Nuclear Factor Erythroid 2-Related Factor 2	A key transcription factor
